# Insecticide-Treated Nets and Protection against Insecticide-Resistant Malaria Vectors in Western Kenya

**DOI:** 10.3201/eid2305.161315

**Published:** 2017-05

**Authors:** Eric Ochomo, Mercy Chahilu, Jackie Cook, Teresa Kinyari, Nabie M. Bayoh, Philippa West, Luna Kamau, Aggrey Osangale, Maurice Ombok, Kiambo Njagi, Evan Mathenge, Lawrence Muthami, Krishanthi Subramaniam, Tessa Knox, Abraham Mnavaza, Martin James Donnelly, Immo Kleinschmidt, Charles Mbogo

**Affiliations:** Kenya Medical Research Institute (KEMRI), Kisumu, Kenya (E. Ochomo, M. Chahilu, A. Osangale, M. Ombok);; London School of Hygiene and Tropical Medicine, London, UK (J. Cook, P. West, I. Kleinschmidt);; University of Nairobi, Nairobi, Kenya (T. Kinyari);; US Centers for Disease Control and Prevention–Kenya, Nairobi (N.M. Bayoh);; KEMRI, Nairobi (L. Kamau, E. Mathenge, L. Muthami);; Ministry of Health, Nairobi (K. Njagi);; Liverpool School of Tropical Medicine, Liverpool, UK (K. Subramaniam, M.J. Donnelly);; World Health Organization, Geneva, Switzerland (T. Knox, A. Mnavaza);; University of Witwatersrand, Johannesburg, South Africa (I. Kleinschmidt);; KEMRI, Kilifi, Kenya (C. Mbogo);; KEMRI–Wellcome Trust, Nairobi (C. Mbogo)

**Keywords:** malaria, malaria infection incidence, insecticide resistance, cohorts, parasites, Kenya, Africa, bed nets, insecticide-treated nets, vector-borne infections, permethrin, deltamethrin

## Abstract

Insecticide resistance might reduce the efficacy of malaria vector control. In 2013 and 2014, malaria vectors from 50 villages, of varying pyrethroid resistance, in western Kenya were assayed for resistance to deltamethrin. Long-lasting insecticide-treated nets (LLIN) were distributed to households at universal coverage. Children were recruited into 2 cohorts, cleared of malaria-causing parasites, and tested every 2 weeks for reinfection. Infection incidence rates for the 2 cohorts were 2.2 (95% CI 1.9–2.5) infections/person-year and 2.8 (95% CI 2.5–3.0) infections/person-year. LLIN users had lower infection rates than non-LLIN users in both low-resistance (rate ratio 0.61, 95% CI 0.42–0.88) and high-resistance (rate ratio 0.55, 95% CI 0.35–0.87) villages (p = 0.63). The association between insecticide resistance and infection incidence was not significant (p = 0.99). Although the incidence of infection was high among net users, LLINs provided significant protection (p = 0.01) against infection with malaria parasite regardless of vector insecticide resistance.

The launch of the Roll Back Malaria (RBM) program in 1998 by the World Health Organization (WHO), United Nations Children’s Fund, United Nations Development Partnership, and the World Bank was a catalyst for renewed global commitment to the fight against malaria, leading to massive investment (*1*). There followed a tremendous decline in disease and death caused by malaria, with a 40% reduction in the incidence of malaria cases between 2000 and 2015 and a reduction in malaria-attributable death from 839,000 in 2000 to 438,000 in 2014 (*1*,*2*). This decline has been brought about principally by the use of insecticide-based vector control tools, such as long-lasting insecticide-treated nets (LLINs) and indoor residual spraying. It is estimated that LLINs have been a key malaria prevention tool in sub-Saharan Africa, accounting for ≈68% of the decline of clinical cases (*3*).

Following the massive scale-up of insecticide-based vector control, resistance was observed in almost all countries in sub-Saharan Africa (http://www.irmapper.com) (*4*). Twelve insecticide products (containing pyrethroids, organochlorines, organophosphates, or carbamates) are available for vector control. Only pyrethroids are used for LLINs because they are safe, efficacious against malaria vectors, and relatively low cost (*5*–*7*).

On a programmatic scale, a 10-fold increase in malaria cases was observed in KwaZulu-Natal, South Africa, subsequent to the re-emergence of pyrethroid-resistant *Anopheles funestus* mosquitoes and emergence of malaria parasite drug resistance to sulfadoxine/pyrimethamine (*8*). Upon switching to DDT for indoor residual spraying and artemether lumefantrine for malaria case management, malaria parasite control was restored with a rapid decline in malaria case incidence (*8*–*10*). Similar observations were made in Uganda, where DDT and pyrethroids were used for indoor residual spraying in the presence of resistance; as soon as carbamates were deployed, the malaria parasite slide positivity rate declined substantially (*11*).

Malaria interventions including universal LLIN coverage, targeted deployment of indoor residual spraying, and prompt diagnosis and treatment have been scaled up in western Kenya since the early 2000s. Control tools targeting endophagic and endophilic malaria vector mosquitoes have been remarkably effective in reducing *An. gambiae* and *An. funestus* mosquito populations that were known to be anthropophilic; these tools have led to lowered malaria inoculation rates and consequently >50% declines in malaria disease and death (*12*–*14*). In western Kenya, malaria prevalence in children <5 years of age declined to ≈30% in 2006, after which it stabilized or slightly increased (*15*,*16*). A possible cause of this persistent infection in children is insecticide resistance in the local vector population. Concerns that resistance could be compromising malaria vector control and, therefore, hampering efforts to lower malaria parasite transmission have led to calls for more effective insecticide resistance management (*17*,*18*).

Population-based active surveillance can complement routine passive sentinel surveillance systems by providing public health data and insights into the complex epidemiology of disease. Active infection-detection cohorts are studies that involve clearing participants of infections, following them up, and testing them at regular intervals, regardless of whether they are symptomatic, until the first infection appears, at which point the follow-up is discontinued. These types of studies provide estimates of time to infection in participants and are useful because they enable estimations of various parameters associated with disease (*19*,*20*). The main advantage of using population-based malaria parasite surveillance is that it provides the data needed to determine the infection rate and the populations at risk for infection (*19*).

This study was implemented as part of a large, multicountry program set up to quantify the impact of insecticide resistance on the effectiveness of insecticide-based vector controls (*21*). To determine if insecticide resistance altered the effectiveness of LLINs in malaria endemic subcounties of western Kenya, we conducted population-based malaria parasite active infection-detection cohort studies.

## Methods

### Study Sites

We conducted this study in 4 malaria-endemic subcounties in western Kenya described previously (*22*). In brief, in 2014, the National Malaria Control Programme conducted a massive campaign to distribute nets; a mix of PermaNet 2.0 (treated with deltamethrin) and Olyset nets (treated with permethrin) were distributed in the 4 subcounties Bondo, Teso, Rachuonyo, and Nyando to meet the universal coverage threshold of 1 net per 2 persons. Subsequent routine distribution was conducted through health facilities to pregnant women and children <5 years of age. Twenty sublocations (hereafter referred to as clusters) were randomly selected from each of the 4 subcounties where the initial insecticide resistance assessment was conducted (*21*,*22*). After the assessment, the clusters were categorized into 3 groups: those with >80% mosquito mortality to deltamethrin or permethrin (categorized as low-resistance clusters), those with mosquito mortality <80% but >60% (categorized as medium-resistance clusters), and those with mosquito mortality <60% (categorized as high-resistance clusters). Finally, 13 low- and high-resistance clusters were selected in Rachuonyo, 11 in Teso, 16 in Bondo, and 10 in Nyando, giving a total of 50 clusters for subsequent studies. Each cluster had 10–30 villages, each with ≈100 households. We recruited children 6–59 months of age from households immediately around larval habitats that were sampled by the entomology teams for assessing insecticide resistance; moving out in concentric circles from the larva habitats, we recruited study participants until 20 eligible and consenting households were enrolled.

### Study Design and Sample Collection

Community health workers were trained to use rapid diagnostic test kits SD Bioline Malaria Ag P.f/Pan (Standard Diagnostics, Gyeonggi-do, South Korea) and CareStart Malaria HRP2 (Pf) (Access Bio, Inc., Somerset, NJ, USA) and to appropriately administer artemisinin combination therapy (Coartem Dispersible [20 mg artemether/120 mg lumefantrine], Novartis, Basel, Switzerland) for the treatment of malaria. The study ran September 2013–May 2014 for cohort 1 and July–December 2014 for cohort 2. Twenty children 6–59 months of age were recruited for each cluster within each cohort. Subject to written informed consent from the parent or caregiver, 1 eligible child was enrolled from each selected household.

At recruitment, all children were treated with a standard therapeutic dose of artemether/lumefantrine. To verify clearance of malaria parasites, 14 days later, thick and thin blood smears were taken from children and assessed for infection by microscopic examination. Any children whose smear results were positive were excluded from follow-up analysis. Community health workers visited each child at home every 2 weeks to test for infection with malaria parasites using rapid diagnostic tests. Children who tested positive for malaria parasite were treated and excluded from further follow-up. LLIN use on the previous night was recorded at each visit. Data in the field was collected using paper forms and then entered into electronic forms made with Microsoft Excel and Access software (Microsoft, Redmond, WA, USA).

From July 2013 through October 2013 and August 2014 through November 2014, we conducted insecticide resistance monitoring in each of the clusters. We collected and reared *An. gambiae* *sensu lato* (*s.l.*) mosquito larvae and adults and tested them for susceptibility to deltamethrin insecticide using the WHO standard test (*22*). We performed these bioassays with both permethrin and deltamethrin at baseline (*22*), but because mortality upon exposure to these 2 insecticides were positively correlated (Technical Appendix Figures 1, 2) and mosquito population size was small, only deltamethrin was used for bioassays in subsequent years. Ethical approval for this study was obtained from the Kenya Medical Research Institute Ethical Review Committee (no. SSC 1677).

### Data Analysis

We used individual visit data for each child to conduct time-to-event analysis to determine incidence rates and incidence rate ratios (RRs) using survival analysis and Poisson regression models. Children who had >5 weeks between visits were censored. Incidence rates and 95% CIs were calculated per person-year for each district and year.

We used insecticide resistance data (percentage mosquito mortality upon exposure to deltamethrin) to dichotomize clusters into high- and low-resistance clusters by using the median mortality for that year, namely, 88% for 2013 (clusters with mortality rates >88% were categorized as low resistance and those with mortality rates <88% as high resistance) and 67% for 2014 (clusters with mortality rates >67% were categorized as low resistance and those with mortality rates <67% as high resistance). In combined analysis of both years, we used the overall median mortality (82%) to dichotomize clusters into high or low resistance for net users and non–net users. Recommended methods (*23*) were used to compute SEs, allowing for the correlation of responses within clusters. We used incidence RRs and corresponding 95% CIs to compare incidence rates between users and nonusers of LLINs and between high- and low-resistance clusters. Modification of the effect of net use on infection incidence depending on insecticide resistance level (mortality to deltamethrin in bioassays) was assessed through the inclusion of an appropriate interaction term in the regression model. Net use was included in models as a time-varying covariate.

We plotted cluster-specific incidence rates for each year and cluster-specific RRs for non–net users and net users against mosquito mortality with deltamethrin exposure. The slope of best-fitting straight lines were determined by using linear regression of cluster-specific incidence on cluster-specific mosquito mortality.

## Results

### Active Infection Cohorts 1 and 2

Approximately 1,000 children were recruited into each active infection cohort. The median age of children at recruitment was 2.5 years for cohort 1 and 2.2 years for cohort 2. For cohort 1, each child was followed for 80 days, and a total of 279 infections were detected; for cohort 2, each child was followed for 95 days, and a total of 483 infections were detected (Table 1). LLIN use was 81.3% for cohort 1 and 85.7% for cohort 2. The overall incidence rate of infection with the malaria parasite was 2.2 (95% CI 1.9–2.5) infections/person-year for cohort 1 and 2.8 (95% CI 2.5–3.0) infections/person-year for cohort 2. The subcounty-specific infection incidences were 1.2–3.0 infections/person-year in cohort 1 and 1.8–4.1 infections/person-year in cohort 2 (Table 2).

**Table 1:** Characteristics of cohorts used to detect active malaria parasite infections, Kenya, 2013 and 2014

Characteristic	Cohort 1, n = 989	Cohort 2, n = 969
Female sex, % (no.)	49 (481)	49 (478)
Median age, y (range, mo–y)	2.5 (4–5)	2.2 (1–6)
Average follow-up per child, d	80	95
No. infections	279	483

**Table 2:** Incidence of malaria parasite infection by subcounty and cohort, Kenya, 2013 and 2014

Subcounty	Cohort	No. clusters	No. children	No. malaria episodes	Total follow-up time, person-years	Incidence, infections/person-year (95% CI)
Bondo	1	16	184	76	35.0	2.2 (1.7–2.7)
	2	16	255	154	58.5	2.6 (2.2–3.1)
Nyando	1	10	147	33	28.3	1.2 (0.8–1.6)
	2	10	180	83	47.3	1.8 (1.4–2.2)
Rachuonyo	1	13	192	97	32.2	3.0 (2.5–3.7)
	2	13	208	136	42.9	3.2 (2.7–3.8)
Teso	1	11	157	73	29.4	2.5 (2.0–3.1)
	2	11	156	110	27.0	4.1 (3.4–4.9)

In low-resistance clusters, the malaria parasite infection incidence rate was 4.0 (95% CI 3.2–5.2) infections/person-year among non–net users and 2.3 (95% CI 2.1–2.5) infections/person-year among net users (RR 0.61, 95% CI 0.42–0.88; p = 0.01). In high-resistance clusters, incidence was 5.3 (95% CI 4.0–7.1) infections/person-year among non–net users and 2.9 (95% CI 1.7–3.2) infections/person-year among net users, a 45% reduction (RR 0.55, 95% CI 0.35–0.87; p = 0.01) in malaria parasite incidence among net users (Table 3).

**Table 3:** Incidence of malaria parasite infection in net users and non–net users in low– and high–insecticide resistance clusters, Kenya, 2013 and 2014

Parameter	No. children	Follow-up time, person-years	No. infections detected	Incidence, infections/person-year (95% CI)	Adjusted RR* (95% CI)	p value
Low resistance (mortality >82%)						
Non–net users	175	15.6	63	4.0 (3.2–5.2)	1.00	
Net users	760	182.9	415	2.3 (2.1–2.5)	0.61 (0.42–0.88)	0.01
High resistance (mortality <82%)						
Non–net users	129	9.0	48	5.3 (4.0–7.1)	1.00	
Net users	772	167.7	494	2.9 (1.7–3.2)	0.55 (0.35–0.87)	0.01
Interaction parameter					0.86 (0.48–1.55)	0.63
Change in incidence per 10% increase in mosquito mortality					0.96 (0.87–1.06)	0.45

*Adjusted for district, year, and visit month.

### Association between Malaria Parasite Infection Incidence and Insecticide Resistance

**Figure 1:**
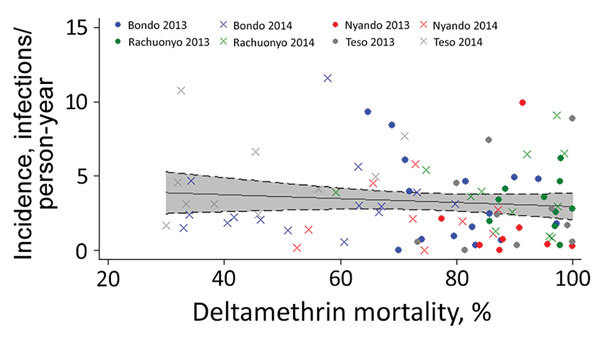
Relationship between deltamethrin insecticide resistance and incidence of malaria parasite infection, 4 subcounties, western Kenya, 2013 and 2014. The incidence of infection in the clusters from subcounties Bondo (blue), Ranchuonya (green), Nyando (red), and Teso (gray) in years 2013 (circles) and 2014 (Xs) were plotted against the corresponding values of mosquito mortality to deltamethrin for that year and that cluster. The best-fit line (with the 95% CI shaded in gray) for the scatterplot is nearly straight, suggesting no relationship between the incidence of infection and *Anopheles gambiae sensu lato* mosquito mortality upon exposure to deltamethrin measured by the World Health Organization bioassay.

We found no association between malaria parasite infection incidence and insecticide resistance when comparing high- and low-resistance clusters. For cohort 1, incidence was 2.2 (95% CI 1.8–2.7) infections/person-year among children living in low-resistance clusters and 2.0 (95% CI 1.6–2.4) infections/person-year among children living in high-resistance clusters (adjusted RR 0.9, 95% CI 0.5–1.6; p = 0.68) (Table 4). For cohort 2, infection incidence was 2.8 (95% CI 2.4–3.2) infections/person-years among children residing in low-resistance clusters and 2.7 (95% CI 2.4–3.1) infections/person-years among children residing in high-resistance clusters (adjusted RR 0.8, 95% CI 0.5–1.2; p = 0.33). After plotting data from 93/100 clusters (data from all subcounties and both years), we found no association between deltamethrin insecticide resistance and malaria parasite infection incidence (Figure 1).

**Table 4:** Incidence of malaria parasite infection in low– and high–insecticide resistance clusters by year, Kenya, 2013 and 2014*

Insecticide resistance	No. children	No. malaria episodes	Total follow-up time, person-years	Incidence, infections/person-year (95% CI)					
Unadjusted		Adjusted	
RR (95% CI)	p value	RR (95% CI)	p value
2013									
Low resistance	290	114	51.6	2.2 (1.8–2.7)	1.0			1.0	
High resistance	311	116	59.2	2.0 (1.6–2.4)	0.9 (0.5–1.6)	0.70		0.9 (0.5–1.6)	0.68
Per 10% increase in mosquito mortality					1.0 (0.7–1.5)	0.99		1.0 (0.7–1.5)	0.98
2014									
Low resistance	433	224	80.7	2.8 (2.4–3.2)	1.0			1.0	
High resistance	460	222	80.9	2.7 (2.4–3.1)	1.0 (0.7–1.4)	0.96		0.8 (0.5–1.2)	0.33
Per 10% increase in mosquito mortality					1.0 (0.9–1.1)	0.90		1.1 (0.9–1.2)	0.24

*In 2013, low resistance was defined as mortality >88% and high resistance as mortality <88%. In 2014, low resistance was defined as mortality >67% and high resistance as mortality <67%. RR, rate ratio.

### Insecticide Resistance

**Figure 2:**
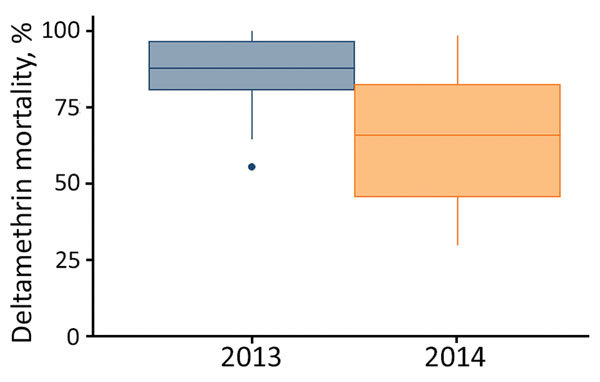
*Anopheles gambiae sensu lato* mosquito mortality to deltamethrin, western Kenya, 2013 and 2014. Mortality was measured using the World Health Organization tube bioassay. Whiskers indicate full range of data; top and bottom lines of boxes indicate 25%–75% interquartile ranges; horizontal lines within boxes indicate medians.

Mosquito mortality ranged 55%–100% in 2013 and 30%–98.5% in 2014. The median (25%–75% interquartile range) mortality rates were 88% (81%–97%) for 2013 and 67% (51%–80%) for 2014 (Figure 2).

### Effect of Insecticide Resistance and Net Use on Malaria Parasite Infection Incidence

The interaction between resistance (high and low) and net use was not significant for either cohort (p = 0.63) (Table 3). The insecticide resistance stratum did not modify the effect of LLIN use on infection incidence.

## Discussion

Because of the widespread use of insecticide-based malaria vector control tools, such as LLINs and indoor residual spraying (*3*,*17*,*24*), insecticide resistance is a rising concern in sub-Saharan Africa (*4*). Our study was designed to estimate the effect that pyrethroid resistance in local malaria vectors had on malaria parasite infection incidence in areas of varying levels of insecticide resistance in western Kenya. Net use was 81.3% in cohort 1 and 85.7% in cohort 2, a small change in net use despite the timing of the LLIN distribution campaign (just before the beginning of cohort 2). Nets were found to be effective at preventing infection in low- and high-resistance clusters. Even with rises in pyrethroid resistance among malaria vectors, nets were shown to be 39% protective in low-resistance clusters and 45% protective in high-resistance clusters. LLINs are still effective in reducing malaria parasite transmission because, aside from the insecticide’s repellent and toxic properties, nets also act as natural barriers that prevent human–vector contact (*25*). Given the positive news that LLINs are still useful in environments with high levels of insecticide resistance, malaria parasite control programs should continue to provide and distribute LLINs and encourage their use in parallel with efforts to develop and evaluate new tools (*18*,*26*).

We did not find a significant association between insecticide resistance and incidence of malaria parasite infection in either year. Concern that insecticide resistance could compromise malaria parasite control has been expressed (*18*,*24*,*27*,*28*), and, with this, the expectation that the incidence of infection would be higher in high-resistance areas. The results of our study, therefore, are surprising, considering the failure some countries have had in malaria vector control after the development of resistance to the insecticides used in indoor residual spraying (*8*,*29*). More specifically, studies have reported resistant mosquitoes surviving exposure to potent nets (nets able to knockdown >80% of susceptible mosquitoes) (*30*,*31*); it was expected that areas with such mosquitoes would have higher malaria parasite infection incidences because the mosquitoes live longer and thus are able to spread malaria parasite for longer.

Several factors might explain why we did not observe a correlation between insecticide resistance and malaria parasite infection incidence. First, as previously mentioned, LLINs serve as a barrier to prevent human–vector contact. If the nets are in good condition and are used consistently and properly, they reduce the chances of mosquito bites and hence malaria parasite transmission (*32*). Second, the WHO tube bioassay does not indicate what level of insecticide resistance is expected to lead to vector control failure, which is a major weakness of the assay (*33*). Therefore, even though we observe insecticide resistance, the mosquito populations might still be susceptible to the toxic effects of the chemical doses used on the nets. This highlights the need for more quantitative methods for monitoring insecticide resistance (*33*,*34*). In addition, a recent study in deltamethrin-resistant mosquitoes showed that sublethal doses of pyrethroids can interfere with parasite development (*35*). Even though these mosquitoes do not succumb to exposure with insecticides, their ability to transmit the malaria parasite is reduced, and therefore, increasing insecticide resistance does not necessarily directly and immediately lead to a major increase in incidence of malaria parasite infection.

However, our results should be interpreted with caution. We have already observed instances of mosquitoes failing to succumb to control tools, such as in a report conducted in the Bungoma district, where resting but still bioactive pyrethroid-resistant *An. gambiae* *sensu stricto* (*s.s.*) mosquitoes were found inside of LLINs without getting killed or repelled (*30*). Also, in Benin, as many as 5 mosquitoes were found to enter damaged LLINs at night (*31*). Similarly, pyrethroid-resistant *An. funestus* mosquitoes have foiled indoor residual spraying efforts to control malaria parasite transmission in South Africa (*8*,*36*).

*An. arabiensis* mosquitoes were the predominant vector in Bondo, Rachuonyo, and Nyando (>90% of the *An. gambiae* *s.l.* population), the other vector being *An. gambiae* *s.s* mosquitoes. In Teso, *An. gambiae* *s.s.* mosquitoes were predominant (>70% of the *An. gambiae* *s.l.* population). It is therefore necessary that, even as programs continue to implement insecticide-based vector control, they follow the guidelines provided by global programs for insecticide resistance management (*28*). Regular insecticide resistance surveillance should continue to be conducted on a wide scale to ensure accurate reporting of the otherwise largely heterogeneous insecticide resistance trends.

Our study had weaknesses that might have affected results, the first being the highly variable nature of the susceptibility data from 1 year to the next and from 1 cluster to the next. As mentioned previously, the WHO tube bioassay is not very informative of the intensity of insecticide resistance. The categorization of net users and non–net users might have substantially confounded results given that net use was not randomly assigned and non–net users were a relatively small number of children who did not prefer to use nets. Last, our study did not consider insecticide resistance in the population *of An. funestus* mosquitoes, a reemerging vector in the region (*37*), mostly because of the difficulty of rearing them in the lab and finding them in larval habitats.

In conclusion, insecticide resistance, especially to pyrethroids, continues to increase in countries in sub-Saharan Africa where LLINs and indoor residual spraying are the mainstays of vector control. The results of this study indicate a utility for continuing LLIN use despite the increasing levels of insecticide resistance in the malaria vector population. However, in our study, even among users of nets, malaria parasite incidence remained alarmingly high. Taken together with other reports suggesting an increase in malaria prevalence in parts of western Kenya with high LLIN coverage (*15*,*16*), the malaria parasite transmission taking place in this region urgently needs to be addressed. Because of their reduced susceptibility, LLINs might not be killing mosquitoes as effectively as they used to. More emphasis needs to be placed on maximizing the coverage and use of LLINs, fully implementing the guidelines on resistance monitoring, and developing more vector control tools to complement existing ones.

Technical Appendix Graph of the relationship between mosquito mortality to permethrin and deltamethrin and an outlier analysis.
