# Supplementary material for: Insecticide-Treated Nets and Protection against Insecticide-Resistant Malaria Vectors in Western Kenya
Source: Emerg Infect Dis. 2017 May;23(5):758–64. doi: 10.3201/eid2305.161315 (PMC5403037; doi:10.3201/eid2305.161315)
Supplement: Technical Appendix — Graph of the relationship between mosquito mortality to permethrin and deltamethrin and an outlier analysis. [file 16-1315-Techapp-s1.pdf]

Article DOI: http://dx.doi.org/10.3201/eid2305.161315

Insecticide-Treated Nets and Protection Against Insecticide-Resistant Malaria Vectors in Western Kenya

Technical Appendix

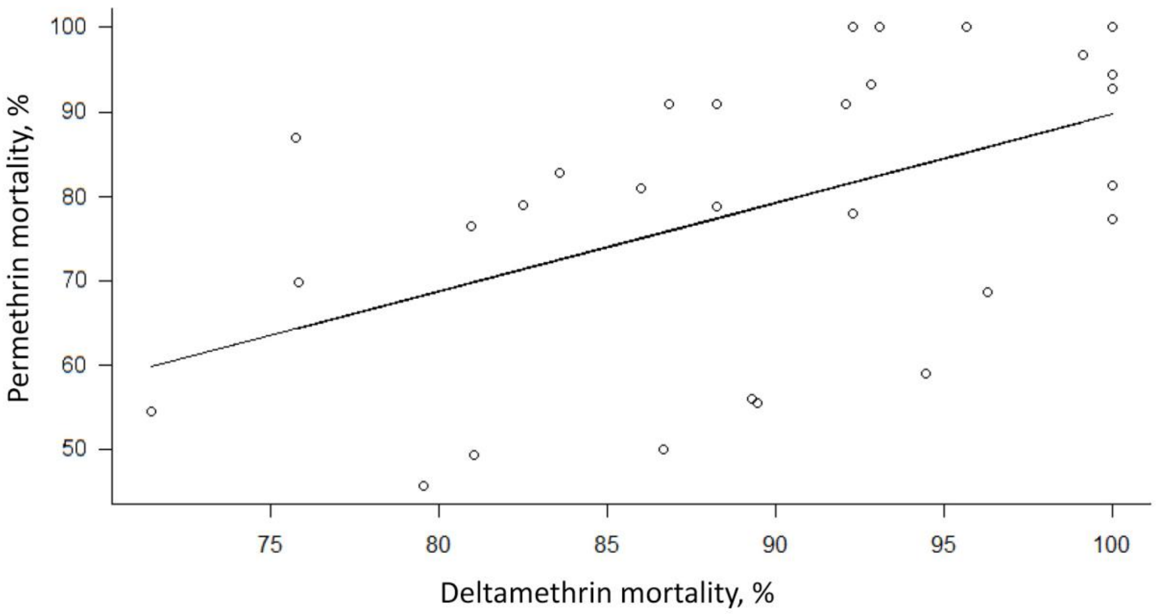

Technical Appendix Figure 1. Correlation between the percentages in permethrin and deltamethrin mortality as determined by the World Health Organization bioassay using *Anopheles arabiensis* collected in western Kenya, 2011. Kendall rank correlation tau = 0.376741; p value = 0.01.

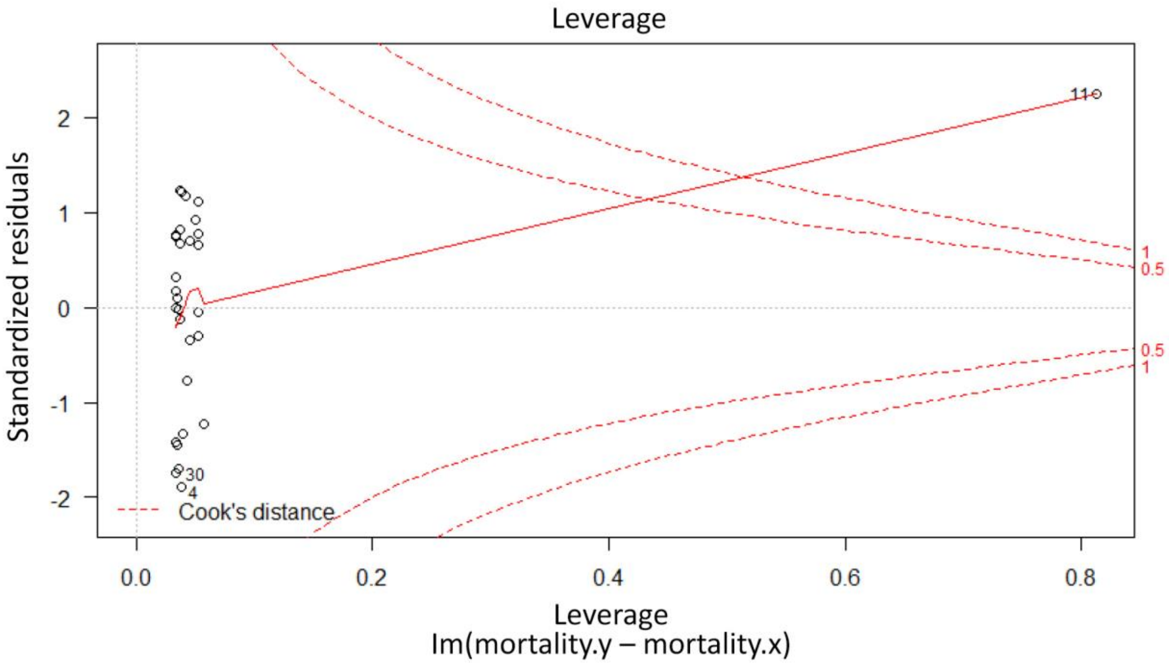

Technical Appendix Figure 2. Plot showing standardized residuals versus leverage. Graph was plotted following removal of an outlier with high leverage (labeled 11) identified by Cook's distance analysis. The Cook's distance for this cluster exceeded the conventional cutoff of 4/n, in which n is the number of observations in the data set.
